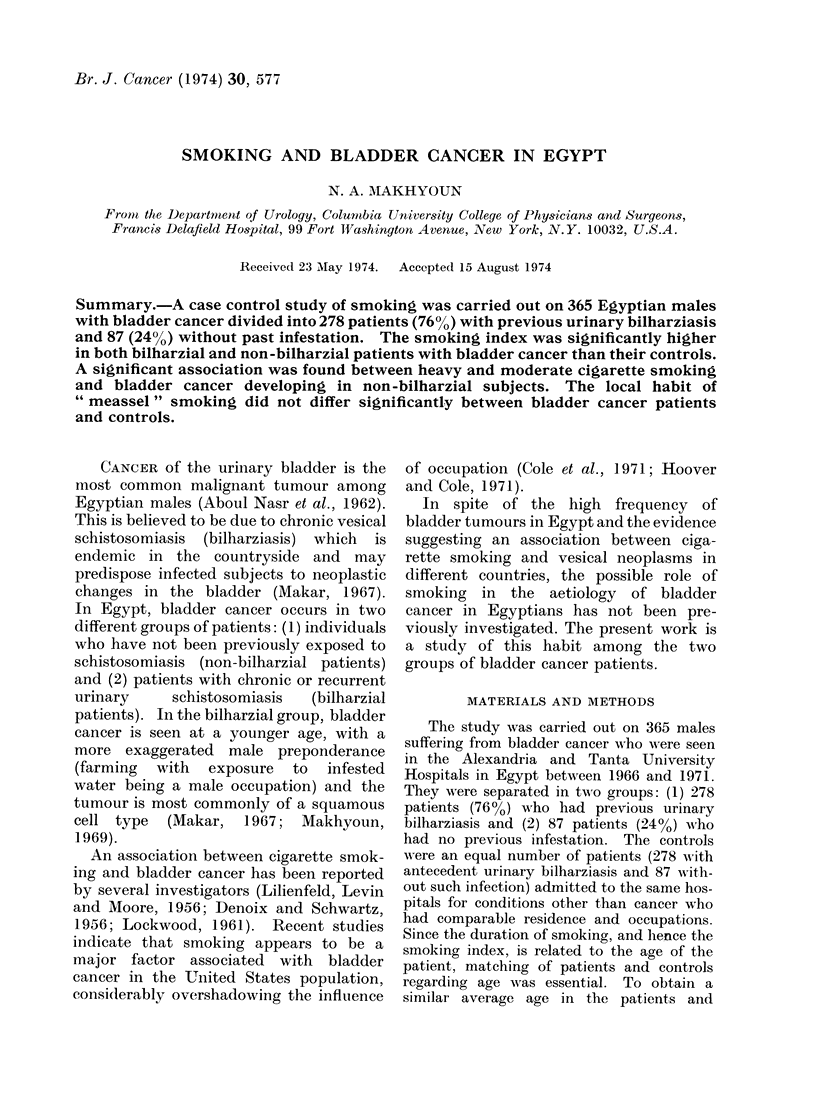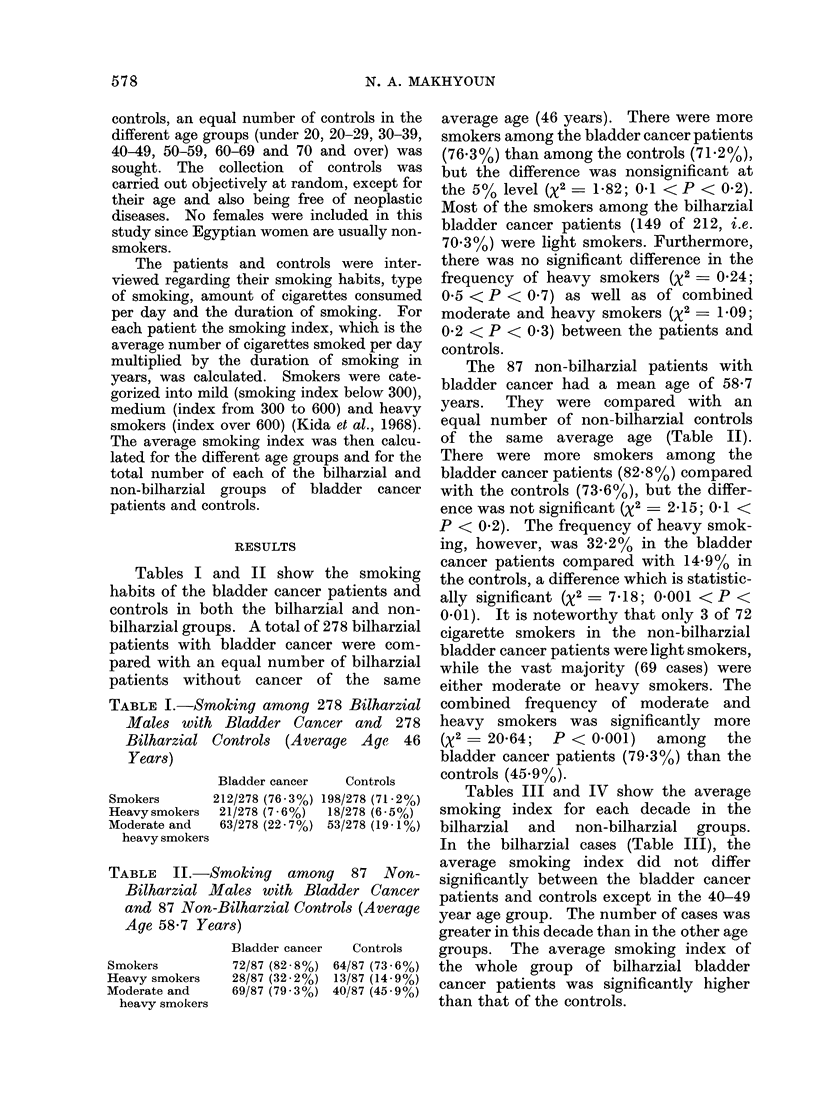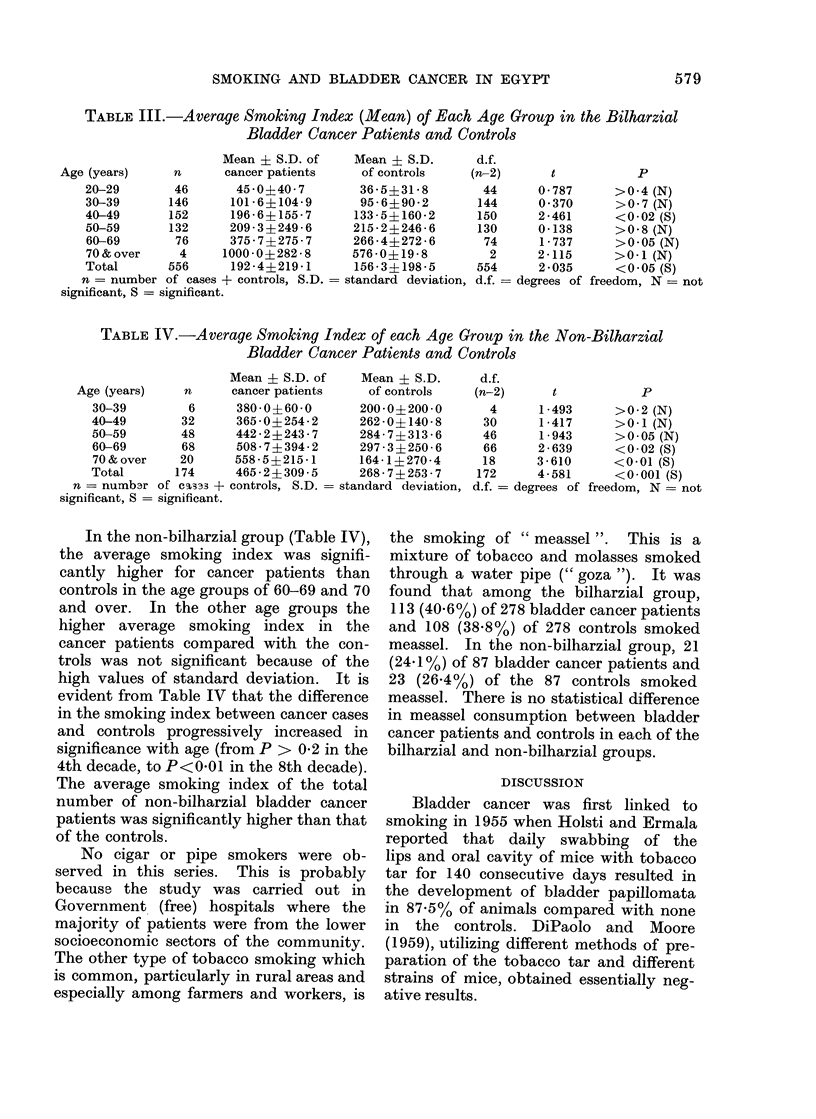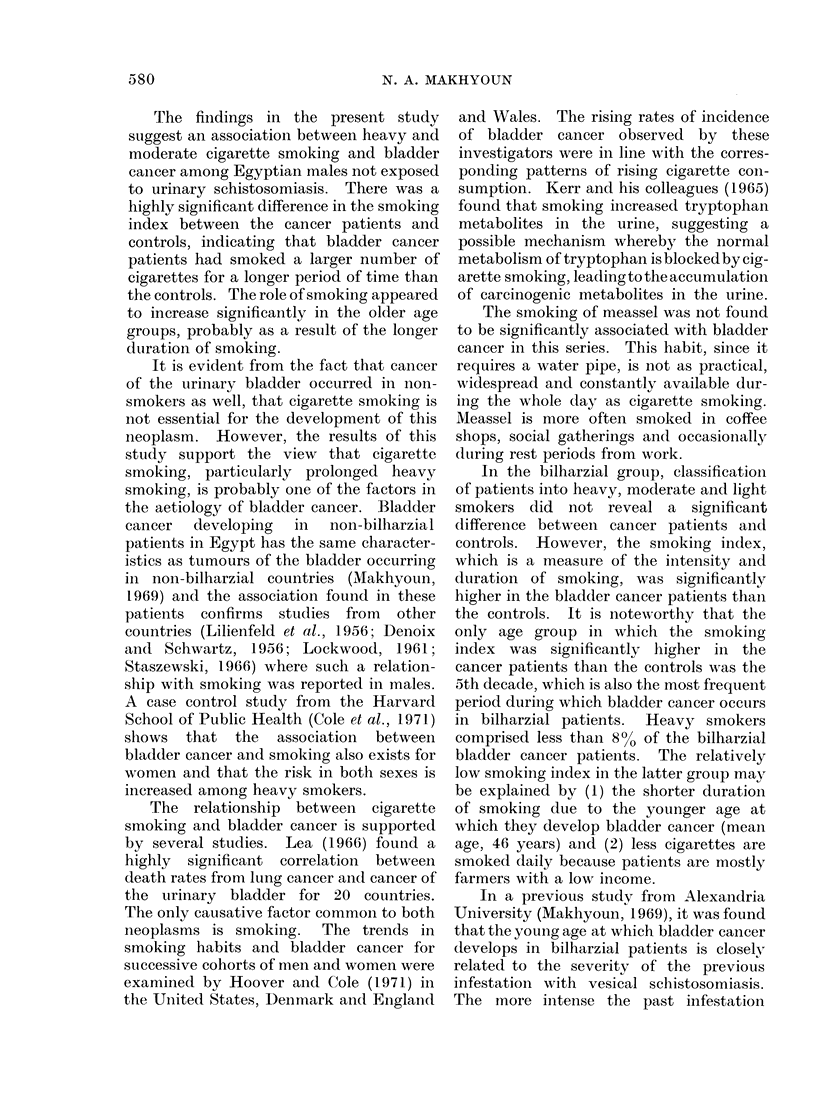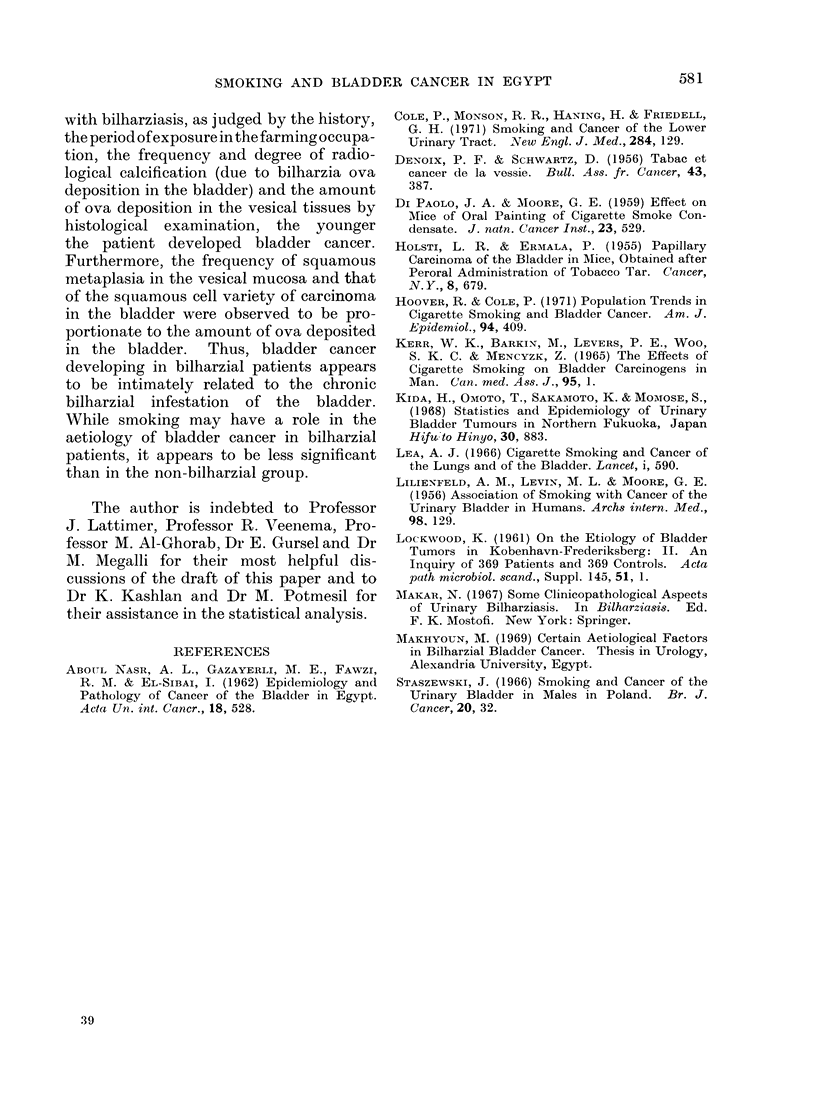# Smoking and Bladder Cancer in Egypt

**DOI:** 10.1038/bjc.1974.238

**Published:** 1974-12

**Authors:** N. A. Makhyoun

## Abstract

A case control study of smoking was carried out on 365 Egyptian males with bladder cancer divided into 278 patients (76%) with previous urinary bilharziasis and 87 (24%) without past infestation. The smoking index was significantly higher in both bilharzial and non-bilharzial patients with bladder cancer than their controls. A significant association was found between heavy and moderate cigarette smoking and bladder cancer developing in non-bilharzial subjects. The local habit of “meassel” smoking did not differ significantly between bladder cancer patients and controls.


*Br. J. Cancer* (1974) **30**, 577

### SMOKING AND BLADDER CANCER IN EGYPT

N. A. MAKHYOUN

*From the Department of Urology, Columbia University College of Physicians and Surgeons, Francis Delafield Hospital, 99 Fort Washington Avenue, New York, N.Y. 10032, U.S.A.*

Received 23 May 1974. Accepted 15 August 1974

**Summary.—A case control study of smoking was carried out on 365 Egyptian males with bladder cancer divided into 278 patients (76%) with previous urinary bilharziasis and 87 (24%) without past infestation. The smoking index was significantly higher in both bilharzial and non-bilharzial patients with bladder cancer than their controls. A significant association was found between heavy and moderate cigarette smoking and bladder cancer developing in non-bilharzial subjects. The local habit of "meassel" smoking did not differ significantly between bladder cancer patients and controls.**

CANCER of the urinary bladder is the most common malignant tumour among Egyptian males (Aboul Nasr *et al.*, 1962). This is believed to be due to chronic vesical schistosomiasis (bilharziasis) which is endemic in the countryside and may predispose infected subjects to neoplastic changes in the bladder (Makar, 1967). In Egypt, bladder cancer occurs in two different groups of patients: (1) individuals who have not been previously exposed to schistosomiasis (non-bilharzial patients) and (2) patients with chronic or recurrent urinary schistosomiasis (bilharzial patients). In the bilharzial group, bladder cancer is seen at a younger age, with a more exaggerated male preponderance (farming with exposure to infested water being a male occupation) and the tumour is most commonly of a squamous cell type (Makar, 1967; Makhyoun, 1969).

An association between cigarette smoking and bladder cancer has been reported by several investigators (Lilienfeld, Levin and Moore, 1956; Denoix and Schwartz, 1956; Lockwood, 1961). Recent studies indicate that smoking appears to be a major factor associated with bladder cancer in the United States population, considerably overshadowing the influence of occupation (Cole *et al.*, 1971; Hoover and Cole, 1971).

In spite of the high frequency of bladder tumours in Egypt and the evidence suggesting an association between cigarette smoking and vesical neoplasms in different countries, the possible role of smoking in the aetiology of bladder cancer in Egyptians has not been previously investigated. The present work is a study of this habit among the two groups of bladder cancer patients.

### MATERIALS AND METHODS

The study was carried out on 365 males suffering from bladder cancer who were seen in the Alexandria and Tanta University Hospitals in Egypt between 1966 and 1971. They were separated in two groups: (1) 278 patients (76%) who had previous urinary bilharziasis and (2) 87 patients (24%) who had no previous infestation. The controls were an equal number of patients (278 with antecedent urinary bilharziasis and 87 without such infection) admitted to the same hospitals for conditions other than cancer who had comparable residence and occupations. Since the duration of smoking, and hence the smoking index, is related to the age of the patient, matching of patients and controls regarding age was essential. To obtain a similar average age in the patients and controls, an equal number of controls in the different age groups (under 20, 20–29, 30–39, 40–49, 50–59, 60–69 and 70 and over) was sought. The collection of controls was carried out objectively at random, except for their age and also being free of neoplastic diseases. No females were included in this study since Egyptian women are usually non-smokers.

The patients and controls were interviewed regarding their smoking habits, type of smoking, amount of cigarettes consumed per day and the duration of smoking. For each patient the smoking index, which is the average number of cigarettes smoked per day multiplied by the duration of smoking in years, was calculated. Smokers were categorized into mild (smoking index below 300), medium (index from 300 to 600) and heavy smokers (index over 600) (Kida *et al.*, 1968). The average smoking index was then calculated for the different age groups and for the total number of each of the bilharzial and non-bilharzial groups of bladder cancer patients and controls.

### RESULTS

Tables I and II show the smoking habits of the bladder cancer patients and controls in both the bilharzial and non-bilharzial groups. A total of 278 bilharzial patients with bladder cancer were compared with an equal number of bilharzial patients without cancer of the same average age (46 years). There were more smokers among the bladder cancer patients (76·3%) than among the controls (71·2%), but the difference was nonsignificant at the 5% level ($\chi^2 = 1{\cdot}82$; $0{\cdot}1 < P < 0{\cdot}2$). Most of the smokers among the bilharzial bladder cancer patients (149 of 212, *i.e.* 70·3%) were light smokers. Furthermore, there was no significant difference in the frequency of heavy smokers ($\chi^2 = 0{\cdot}24$; $0{\cdot}5 < P < 0{\cdot}7$) as well as of combined moderate and heavy smokers ($\chi^2 = 1{\cdot}09$; $0{\cdot}2 < P < 0{\cdot}3$) between the patients and controls.

TABLE I.—*Smoking among 278 Bilharzial Males with Bladder Cancer and 278 Bilharzial Controls (Average Age 46 Years)*

| | Bladder cancer | Controls |
|---|---|---|
| Smokers | 212/278 (76·3%) | 198/278 (71·2%) |
| Heavy smokers | 21/278 (7·6%) | 18/278 (6·5%) |
| Moderate and heavy smokers | 63/278 (22·7%) | 53/278 (19·1%) |

TABLE II.—*Smoking among 87 Non-Bilharzial Males with Bladder Cancer and 87 Non-Bilharzial Controls (Average Age 58·7 Years)*

| | Bladder cancer | Controls |
|---|---|---|
| Smokers | 72/87 (82·8%) | 64/87 (73·6%) |
| Heavy smokers | 28/87 (32·2%) | 13/87 (14·9%) |
| Moderate and heavy smokers | 69/87 (79·3%) | 40/87 (45·9%) |

The 87 non-bilharzial patients with bladder cancer had a mean age of 58·7 years. They were compared with an equal number of non-bilharzial controls of the same average age (Table II). There were more smokers among the bladder cancer patients (82·8%) compared with the controls (73·6%), but the difference was not significant ($\chi^2 = 2{\cdot}15$; $0{\cdot}1 < P < 0{\cdot}2$). The frequency of heavy smoking, however, was 32·2% in the bladder cancer patients compared with 14·9% in the controls, a difference which is statistically significant ($\chi^2 = 7{\cdot}18$; $0{\cdot}001 < P < 0{\cdot}01$). It is noteworthy that only 3 of 72 cigarette smokers in the non-bilharzial bladder cancer patients were light smokers, while the vast majority (69 cases) were either moderate or heavy smokers. The combined frequency of moderate and heavy smokers was significantly more ($\chi^2 = 20{\cdot}64$; $P < 0{\cdot}001$) among the bladder cancer patients (79·3%) than the controls (45·9%).

Tables III and IV show the average smoking index for each decade in the bilharzial and non-bilharzial groups. In the bilharzial cases (Table III), the average smoking index did not differ significantly between the bladder cancer patients and controls except in the 40–49 year age group. The number of cases was greater in this decade than in the other age groups. The average smoking index of the whole group of bilharzial bladder cancer patients was significantly higher than that of the controls.

TABLE III.—*Average Smoking Index (Mean) of Each Age Group in the Bilharzial Bladder Cancer Patients and Controls*

| Age (years) | $n$ | Mean ± S.D. of cancer patients | Mean ± S.D. of controls | d.f. ($n$–2) | $t$ | $P$ |
|---|---|---|---|---|---|---|
| 20–29 | 46 | 45·0±40·7 | 36·5±31·8 | 44 | 0·787 | >0·4 (N) |
| 30–39 | 146 | 101·6±104·9 | 95·6±90·2 | 144 | 0·370 | >0·7 (N) |
| 40–49 | 152 | 196·6±155·7 | 133·5±160·2 | 150 | 2·461 | <0·02 (S) |
| 50–59 | 132 | 209·3±249·6 | 215·2±246·6 | 130 | 0·138 | >0·8 (N) |
| 60–69 | 76 | 375·7±275·7 | 266·4±272·6 | 74 | 1·737 | >0·05 (N) |
| 70 & over | 4 | 1000·0±282·8 | 576·0±19·8 | 2 | 2·115 | >0·1 (N) |
| Total | 556 | 192·4±219·1 | 156·3±198·5 | 554 | 2·035 | <0·05 (S) |

$n$ = number of cases + controls, S.D. = standard deviation, d.f. = degrees of freedom, N = not significant, S = significant.

TABLE IV.—*Average Smoking Index of each Age Group in the Non-Bilharzial Bladder Cancer Patients and Controls*

| Age (years) | $n$ | Mean ± S.D. of cancer patients | Mean ± S.D. of controls | d.f. ($n$–2) | $t$ | $P$ |
|---|---|---|---|---|---|---|
| 30–39 | 6 | 380·0±60·0 | 200·0±200·0 | 4 | 1·493 | >0·2 (N) |
| 40–49 | 32 | 365·0±254·2 | 262·0±140·8 | 30 | 1·417 | >0·1 (N) |
| 50–59 | 48 | 442·2±243·7 | 284·7±313·6 | 46 | 1·943 | >0·05 (N) |
| 60–69 | 68 | 508·7±394·2 | 297·3±250·6 | 66 | 2·639 | <0·02 (S) |
| 70 & over | 20 | 558·5±215·1 | 164·1±270·4 | 18 | 3·610 | <0·01 (S) |
| Total | 174 | 465·2±309·5 | 268·7±253·7 | 172 | 4·581 | <0·001 (S) |

$n$ = number of cases + controls, S.D. = standard deviation, d.f. = degrees of freedom, N = not significant, S = significant.

In the non-bilharzial group (Table IV), the average smoking index was significantly higher for cancer patients than controls in the age groups of 60–69 and 70 and over. In the other age groups the higher average smoking index in the cancer patients compared with the controls was not significant because of the high values of standard deviation. It is evident from Table IV that the difference in the smoking index between cancer cases and controls progressively increased in significance with age (from $P > 0{\cdot}2$ in the 4th decade, to $P < 0{\cdot}01$ in the 8th decade). The average smoking index of the total number of non-bilharzial bladder cancer patients was significantly higher than that of the controls.

No cigar or pipe smokers were observed in this series. This is probably because the study was carried out in Government (free) hospitals where the majority of patients were from the lower socioeconomic sectors of the community. The other type of tobacco smoking which is common, particularly in rural areas and especially among farmers and workers, is the smoking of " meassel ". This is a mixture of tobacco and molasses smoked through a water pipe (" goza "). It was found that among the bilharzial group, 113 (40·6%) of 278 bladder cancer patients and 108 (38·8%) of 278 controls smoked meassel. In the non-bilharzial group, 21 (24·1%) of 87 bladder cancer patients and 23 (26·4%) of the 87 controls smoked meassel. There is no statistical difference in meassel consumption between bladder cancer patients and controls in each of the bilharzial and non-bilharzial groups.

### DISCUSSION

Bladder cancer was first linked to smoking in 1955 when Holsti and Ermala reported that daily swabbing of the lips and oral cavity of mice with tobacco tar for 140 consecutive days resulted in the development of bladder papillomata in 87·5% of animals compared with none in the controls. DiPaolo and Moore (1959), utilizing different methods of preparation of the tobacco tar and different strains of mice, obtained essentially negative results.

The findings in the present study suggest an association between heavy and moderate cigarette smoking and bladder cancer among Egyptian males not exposed to urinary schistosomiasis. There was a highly significant difference in the smoking index between the cancer patients and controls, indicating that bladder cancer patients had smoked a larger number of cigarettes for a longer period of time than the controls. The role of smoking appeared to increase significantly in the older age groups, probably as a result of the longer duration of smoking.

It is evident from the fact that cancer of the urinary bladder occurred in non-smokers as well, that cigarette smoking is not essential for the development of this neoplasm. However, the results of this study support the view that cigarette smoking, particularly prolonged heavy smoking, is probably one of the factors in the aetiology of bladder cancer. Bladder cancer developing in non-bilharzial patients in Egypt has the same characteristics as tumours of the bladder occurring in non-bilharzial countries (Makhyoun, 1969) and the association found in these patients confirms studies from other countries (Lilienfeld *et al.*, 1956; Denoix and Schwartz, 1956; Lockwood, 1961; Staszewski, 1966) where such a relationship with smoking was reported in males. A case control study from the Harvard School of Public Health (Cole *et al.*, 1971) shows that the association between bladder cancer and smoking also exists for women and that the risk in both sexes is increased among heavy smokers.

The relationship between cigarette smoking and bladder cancer is supported by several studies. Lea (1966) found a highly significant correlation between death rates from lung cancer and cancer of the urinary bladder for 20 countries. The only causative factor common to both neoplasms is smoking. The trends in smoking habits and bladder cancer for successive cohorts of men and women were examined by Hoover and Cole (1971) in the United States, Denmark and England and Wales. The rising rates of incidence of bladder cancer observed by these investigators were in line with the corresponding patterns of rising cigarette consumption. Kerr and his colleagues (1965) found that smoking increased tryptophan metabolites in the urine, suggesting a possible mechanism whereby the normal metabolism of tryptophan is blocked by cigarette smoking, leading to the accumulation of carcinogenic metabolites in the urine.

The smoking of meassel was not found to be significantly associated with bladder cancer in this series. This habit, since it requires a water pipe, is not as practical, widespread and constantly available during the whole day as cigarette smoking. Meassel is more often smoked in coffee shops, social gatherings and occasionally during rest periods from work.

In the bilharzial group, classification of patients into heavy, moderate and light smokers did not reveal a significant difference between cancer patients and controls. However, the smoking index, which is a measure of the intensity and duration of smoking, was significantly higher in the bladder cancer patients than the controls. It is noteworthy that the only age group in which the smoking index was significantly higher in the cancer patients than the controls was the 5th decade, which is also the most frequent period during which bladder cancer occurs in bilharzial patients. Heavy smokers comprised less than 8% of the bilharzial bladder cancer patients. The relatively low smoking index in the latter group may be explained by (1) the shorter duration of smoking due to the younger age at which they develop bladder cancer (mean age, 46 years) and (2) less cigarettes are smoked daily because patients are mostly farmers with a low income.

In a previous study from Alexandria University (Makhyoun, 1969), it was found that the young age at which bladder cancer develops in bilharzial patients is closely related to the severity of the previous infestation with vesical schistosomiasis. The more intense the past infestation with bilharziasis, as judged by the history, the period of exposure in the farming occupation, the frequency and degree of radiological calcification (due to bilharzia ova deposition in the bladder) and the amount of ova deposition in the vesical tissues by histological examination, the younger the patient developed bladder cancer. Furthermore, the frequency of squamous metaplasia in the vesical mucosa and that of the squamous cell variety of carcinoma in the bladder were observed to be proportionate to the amount of ova deposited in the bladder. Thus, bladder cancer developing in bilharzial patients appears to be intimately related to the chronic bilharzial infestation of the bladder. While smoking may have a role in the aetiology of bladder cancer in bilharzial patients, it appears to be less significant than in the non-bilharzial group.

The author is indebted to Professor J. Lattimer, Professor R. Veenema, Professor M. Al-Ghorab, Dr E. Gursel and Dr M. Megalli for their most helpful discussions of the draft of this paper and to Dr K. Kashlan and Dr M. Potmesil for their assistance in the statistical analysis.

### REFERENCES

Aboul Nasr, A. L., Gazayerli, M. E., Fawzi, R. M. & El-Sibai, I. (1962) Epidemiology and Pathology of Cancer of the Bladder in Egypt. *Acta Un. int. Cancr.*, **18**, 528.

Cole, P., Monson, R. R., Haning, H. & Friedell, G. H. (1971) Smoking and Cancer of the Lower Urinary Tract. *New Engl. J. Med.*, **284**, 129.

Denoix, P. F. & Schwartz, D. (1956) Tabac et cancer de la vessie. *Bull. Ass. fr. Cancer*, **43**, 387.

Di Paolo, J. A. & Moore, G. E. (1959) Effect on Mice of Oral Painting of Cigarette Smoke Condensate. *J. natn. Cancer Inst.*, **23**, 529.

Holsti, L. R. & Ermala, P. (1955) Papillary Carcinoma of the Bladder in Mice, Obtained after Peroral Administration of Tobacco Tar. *Cancer, N.Y.*, **8**, 679.

Hoover, R. & Cole, P. (1971) Population Trends in Cigarette Smoking and Bladder Cancer. *Am. J. Epidemiol.*, **94**, 409.

Kerr, W. K., Barkin, M., Levers, P. E., Woo, S. K. C. & Mencyzk, Z. (1965) The Effects of Cigarette Smoking on Bladder Carcinogens in Man. *Can. med. Ass. J.*, **95**, 1.

Kida, H., Omoto, T., Sakamoto, K. & Momose, S., (1968) Statistics and Epidemiology of Urinary Bladder Tumours in Northern Fukuoka, Japan *Hifu to Hinyo*, **30**, 883.

Lea, A. J. (1966) Cigarette Smoking and Cancer of the Lungs and of the Bladder. *Lancet*, i, 590.

Lilienfeld, A. M., Levin, M. L. & Moore, G. E. (1956) Association of Smoking with Cancer of the Urinary Bladder in Humans. *Archs intern. Med.*, **98**, 129.

Lockwood, K. (1961) On the Etiology of Bladder Tumors in Kobenhavn-Frederiksberg: II. An Inquiry of 369 Patients and 369 Controls. *Acta path microbiol. scand.*, Suppl. 145, **51**, 1.

Makar, N. (1967) Some Clinicopathological Aspects of Urinary Bilharziasis. In *Bilharziasis*. Ed. F. K. Mostofi. New York: Springer.

Makhyoun, M. (1969) Certain Aetiological Factors in Bilharzial Bladder Cancer. Thesis in Urology, Alexandria University, Egypt.

Staszewski, J. (1966) Smoking and Cancer of the Urinary Bladder in Males in Poland. *Br. J. Cancer*, **20**, 32.